# Reframing Pandemic Preparedness: Why Positive-Pressure Exposure Control Merits Formal Public Health Evaluation

**DOI:** 10.2196/90211

**Published:** 2026-08-27

**Authors:** Yusaku Fujii

**Affiliations:** 1 Graduate School of Science and Technology, Gunma University, 1-5-1 Tenjin-cho, Kiryu, Gunma, 3768515, Japan, 81 08035505585

**Keywords:** pandemic preparedness, powered air-purifying respirator, PAPR, positive-pressure exposure control, population-level intervention, public health evaluation, privacy-preserving governance

## Abstract

Powered air-purifying respirators (PAPRs) are respiratory protective devices that actively supply filtered air and maintain positive pressure in the breathing zone, thereby limiting the inward leakage of unfiltered ambient air through gaps. Their principles and technology are already established. Positive-pressure PAPRs have primarily been used by health care workers in high-risk environments. However, their potential as an individual-level infection-control measure applicable to the general public has not been formally positioned for evaluation within the context of pandemic preparedness. This viewpoint aims to demonstrate the rationale for and need to position exposure control using positive-pressure PAPRs designed for the general public as a subject of systematic public health evaluation. Unlike existing measures that depend primarily on behavioral change, spatial separation, or acquisition of immunity, this intervention directly reduces aerosol inhalation exposure in an individual’s breathing zone—the physical process that precedes airborne infection. Existing engineering studies increasingly make it possible to consider a public-oriented positive-pressure PAPR not merely as an idea but as a concrete intervention model with the potential for manufacture and implementation. However, the extent to which engineering measures of aerosol protection translate into individual-level prevention of infection and population-level infection control under real-world conditions must be established empirically. Individual-level preventive effectiveness, population-level infection-control effectiveness, engineering performance, feasibility of implementation, sustained use, social acceptability, cost, and equity must therefore be evaluated comprehensively by integrating medical and engineering assessments within a public health framework. This article does not advocate the immediate introduction of positive-pressure PAPRs for the general public. Rather, it identifies an institutional gap: established principles and technologies have not been incorporated into formal public health evaluation as a population-level infection-control measure. Evaluating positive-pressure exposure control for the general public is necessary to determine whether it could broaden policy options in future respiratory infectious disease pandemics and reduce excessive reliance on measures with major societal consequences.

## Introduction

Two broad strategies played major roles in the global response to the COVID-19 pandemic [1]. The first combined behavioral interventions, such as mask mandates and physical distancing [2], with mass vaccination intended to establish population immunity [3]. The second was lockdown—the broad restriction of social and economic activity when health systems faced the threat of collapse [4].

Although lockdowns helped curb the spread of infection [4], the COVID-19 pandemic and the accompanying broad societal restrictions placed severe burdens on economies, education, and social life [5,6]. Lessons from the COVID-19 pandemic have reaffirmed the importance of infection surveillance, early-response systems, international cooperation frameworks, and national pandemic preparedness plans [7,8]. At the same time, reconciling epidemic control with the continuation of social activity requires not only the continued improvement of existing measures but also the exploration and evaluation, during nonemergency periods, of new interventions that could complement them.

Existing measures include means of physically reducing inhalation exposure, such as masks. However, measures intended for the general public that actively supply filtered air, maintain positive pressure in the breathing zone, limit the inward leakage of unfiltered ambient air, and control inhalation exposure with a high protection factor have not been established as formal public health options. During respiratory infectious disease pandemics, inhalation of infectious aerosols can be a major route of transmission [9,10]. Because infection is influenced by the infectious dose reaching the respiratory tract, substantially reducing that dose below the threshold could, in theory, prevent infection regardless of the ambient pathogen concentration [11].

A potential third axis is an engineering intervention that directly and physically reduces inhalation exposure. One concrete means is the powered air-purifying respirator (PAPR). A positive-pressure PAPR actively supplies filtered air and maintains positive pressure in the breathing zone, thereby limiting the inward leakage of unfiltered ambient air through gaps. Its principles and technology are already established, and PAPRs have primarily been used in high-risk clinical settings, such as intensive care units and infectious disease isolation environments. However, their potential as an individual-level infection-control measure applicable to the general public has not been formally evaluated as a public health option for pandemic preparedness [12].

The author has named the concept of exposure control using positive-pressure PAPRs for the general public “PAPR for Everyone” (PFE). The concept was proposed at the beginning of the pandemic in 2020 [13], after which the engineering and epidemiological evidence underlying it was accumulated in stages [14-18]. Since 2024, findings concerning manufacturability and ease of implementation, required use rates, respiratory-state estimation, real-time leak detection, and time-managed use have been published in journals including *JMIR Biomedical Engineering* and *Scientific Reports*. These engineering and theoretical studies have brought positive-pressure PAPRs for the general public to a stage at which they can be considered a concrete, testable intervention rather than merely a conceptual proposal.

This article aims to demonstrate the rationale and need for positioning exposure control using positive-pressure PAPRs for the general public as a subject of formal public health evaluation in pandemic preparedness. It summarizes the engineering and theoretical evidence accumulated for PFE and examines why this option has been overlooked. Rather than calling for its immediate introduction, we propose beginning with field studies during seasonal influenza epidemics and systematically evaluating individual-level preventive effectiveness, population-level infection-control effectiveness, engineering performance, feasibility of implementation, sustained use, social acceptability, cost, and equity by integrating medical and engineering assessments within a public health framework.

## Current Evidence

### Dose-Based Infection Control and the Significance of PAPRs

Infection by airborne pathogens requires inhalation of a sufficient quantity of viable pathogens—an infectious dose [11,19]. Interventions that reduce this dose below the relevant threshold reduce the probability of infection [11,19]. Accordingly, a means of directly and reliably reducing inhalation exposure could, in principle, be a highly powerful infection-control intervention [11,19].

High-efficiency particulate air (HEPA) filters have a collection efficiency of at least 99.9% for 0.3 μm test particles, while the most penetrating particle size (MPPS) varies around 0.3 μm depending on the filter medium, flow rate, and other conditions [20]. A PAPR uses a blower to actively supply air cleaned through a HEPA filter to the breathing zone and prevents the ingress of unfiltered ambient air by maintaining positive pressure in that zone [14,21]. The assigned protection factor (APF) for a helmet- or hood-type PAPR is generally 25; however, an APF of 1000 may be applied to models for which the manufacturer demonstrates performance of at least 1000 based on a workplace protection factor (WPF), simulated workplace protection factor (SWPF), or equivalent test [22]. This article focuses on positive-pressure PAPRs with an APF of 1000 that meet these testing requirements. Under specified conditions of fit and operation, they are expected to reduce the concentration of inhaled aerosols to approximately one-thousandth of the ambient concentration [22]. A reduction in inhaled infectious dose of this magnitude would be expected to substantially reduce the probability of infection in ordinary living environments [11,19].

This protective mechanism differs fundamentally from that of passive masks and negative-pressure respirators. In such devices, inhalation creates negative pressure inside the device, and inadequate facial sealing may allow unfiltered ambient air to enter through gaps. By contrast, a PAPR actively maintains positive pressure in the breathing zone and therefore limits inward leakage through gaps in principle. Comparing an APF of 10 for a negative-pressure respirator with an APF of 1000 for a positive-pressure PAPR, inhalation exposure under specified conditions of fit and operation differs by approximately 100-fold, or about 2 orders of magnitude [22]. This substantial reduction in inhaled infectious dose could be expected to have a decisive effect, bringing the probability of infection close to zero in ordinary living environments.

Cost is one barrier to widespread use. Medical-grade PAPRs are expensive, although their basic structure consists only of a nonwoven filter, pump, and battery [21,23]. The author demonstrated that a prototype providing equivalent performance could be assembled from commercially available components costing approximately US $40 in total [14]. Mass production is highly likely to reduce costs to a level suitable for public health procurement.

### Findings From the Author’s Research Group

The author’s research group has progressively developed the engineering and epidemiological foundations of PFE. Four studies make important contributions.

First, the rate of PAPR use required for it to function as an alternative to lockdown was quantified using an epidemiological mathematical model based on the effective reproduction number [15]. Simulations showed that continuous use by approximately 55% or more of the population could reduce the effective reproduction number from 2 to <0.9. They also showed that, if everyone used a PAPR, an approximately 55% reduction in each individual’s probability of infection would produce an equivalent population-level infection-control effect (R_t_).

Second, a detailed fluid-flow model of airflow inside a PAPR was developed [16]. A method was proposed for estimating respiratory state from a differential-pressure sensor and a pulse-width modulation (PWM) control signal, enabling respiratory-phase-adaptive control intended to provide pressure-supported breathing through reverse-pressure control.

Third, validation with a physical prototype demonstrated that the wearer’s respiratory flow could be estimated accurately in real time and that leakage associated with improper fit could be detected using only 1 differential-pressure sensor and the pump drive signal [17]. These findings established a technical basis for respiratory-state-responsive airflow control and the implementation of pressure-supported breathing.

Finally, the PAPR Wear-Rate Management System Network (PWS-NET) was proposed and prototyped [18]. This system combines real-time use and leak detection using a differential-pressure sensor, GPS location reporting through a smartphone, and a rule-based web server. It introduces the concept of saved allowable time (SAT), defined as the amount of time during which an individual is permitted to remove the PAPR. Prototype validation confirmed that SAT updating, violation detection, and real-time feedback to users functioned as intended.

PWS-NET is a framework that simultaneously enables population-wide monitoring of use rates and individual selection of SAT [18]. In this context, an AI-output governance framework comprising the Guide to the Expression of Legitimacy of Output (GLO) and the Verifiable Record of AI Output (VRAIO) becomes important [24]. GLO is a common language that treats an output candidate generated by an AI system as a “claim” and uses structured metadata to express whether its purpose and content comply with publicly disclosed rules. In this article, the same concept is envisaged as applying to the use of information and system decisions in PWS-NET. VRAIO provides the institutional and technical infrastructure needed to ensure the authenticity and verifiability of GLO declarations through compliance verification by an independent recorder, tamper-resistant records, and subsequent independent audits [24].

The more effective PWS-NET becomes, the greater its capacity to collect and use personal information. The GLO-VRAIO framework could impose verifiable institutional constraints on that capacity and offer a promising governance option for implementing PFE not as an instrument of intensified surveillance, but as public health infrastructure premised on privacy protection. In other words, a 3-layer structure—physical protection by PAPRs, use-rate management by PWS-NET, and governance of information use and system outputs through GLO-VRAIO—would seek to reconcile effective infection control with a high level of protection for citizens’ rights.

### Logical Implications of the Current Evidence

Four implications follow from the aforementioned analysis. First, during pandemics dominated by airborne transmission, PAPRs could partially substitute for lockdown and may make it possible to manage infection risk without halting societal functions. Second, positive-pressure exposure control using PAPRs directly controls the physical process of inhalation exposure to infectious aerosols, irrespective of pathogen type. It may therefore be available immediately after the emergence of a new respiratory pathogen, without waiting for pathogen-specific vaccines or therapeutics. If inhalation exposure can be reduced sufficiently, it may be possible to control transmission without relying solely on acquisition of immunity and to secure time for pathogen identification, characterization of transmission, and development of vaccines and therapeutics. Third, PAPRs give individuals the option to reduce their risk of infection, and voluntary uptake could contribute to society-wide epidemic control. Finally, combining PWS-NET with the GLO-VRAIO framework may enable the information needed for infection control to be used while making it possible to verify the purposes for which information on use status, movement histories, and biometric information is collected and analyzed, as well as compliance with privacy protection rules [18,24]. This could provide an institutional basis for reconciling infection control grounded in the principle that “what cannot be measured cannot be controlled” with the protection of citizens’ privacy and rights.

These implications indicate that PFE could become an additional axis of pandemic response, linking individual-level inhalation-exposure control to population-level infection control [14,15,18].

## Scope for Improvement: PAPR Technology Has Yet to Be Fully Optimized

Current PAPRs are used primarily in specialized hazardous environments in medical and industrial settings. They are produced in small quantities for limited markets and are subject to rigorous performance testing, quality assurance, and certification. Consequently, they tend to be expensive, and there has been little product-development competition concerning the lightness, comfort, functionality, design, and affordability required for routine use by the general public [23,25]. At the same time, their basic functional components are a nonwoven filter, an electric fan, and a battery, while their hoods or helmets are composed mainly of plastic, fabric, or nonwoven materials [14,21,23]. Current prices and forms therefore partly reflect conventional applications and market structures rather than inherent technological limitations.

Because an integrated helmet-type PAPR contains a battery, electric fan, control circuitry, and supporting structure, it also offers a suitable platform for integrating sensors that measure respiratory state and leakage, communication functions, augmented-reality displays, and smartphone functions. However, the required protective performance and comfort must be reconciled while evaluating the effects of added functions on weight, power consumption, heat generation, safety, and privacy. In addition to helmet-type devices, possible implementations of positive-pressure exposure control include personal booth systems for offices and restaurants [14,15] and ventilation-unit replacement systems that cover entire interiors of homes, offices, hotels, and other buildings.

This article has focused primarily on positive-pressure PAPRs with an APF of 1000 as a benchmark for high individual protection. However, the performance required of PAPRs for the general public need not uniformly be an APF of 1000. A lower protection factor may suffice if it can achieve the individual-level objective of keeping the inhaled infectious dose below the level required to establish infection or the public health objective of maintaining the effective reproduction number below 1 across the population. The required performance level will vary with the pathogen’s infectious dose, environmental concentration, duration of exposure, rate of PAPR use, and continuity of use. Future empirical studies should therefore verify the performance of APF 1000-class devices while also identifying the protection level that is necessary and sufficient to achieve individual- and population-level infection-control objectives.

PFE can be deployed without PWS-NET; PWS-NET is a future option intended to reconcile use-rate management with individual choice of SAT [18]. However, the use of wearing histories, location information, proximity information, and biometric data could permit inferences about an individual’s patterns of daily life. By using GLO to make structured declarations of the purposes of data acquisition and analysis and VRAIO to verify, record, and audit compliance with privacy protection rules, information use by PWS-NET could be handled as a verifiable institutional procedure [24].

If positive-pressure PAPRs for the general public become a formal subject of public health evaluation and future demand becomes clear, mass production, component standardization, and competition among products may advance, promoting lower prices and designs suitable for daily use. The present generation of prototypes is a starting point, not a finished product. By continuously incorporating public health needs and evaluation results into engineering development, PAPRs must evolve into lighter, more comfortable, and safer devices while maintaining the required protective performance.

In the longer term, if PAPRs become sufficiently comfortable and convenient, people may voluntarily choose them to breathe purified air regardless of whether governments request their use. Just as people routinely use hygienically managed water, this concept envisages a future in which individuals can also choose purified air to breathe. When PAPRs shift from specialized protective equipment for emergencies to routine devices for controlling inhalation exposure, control of airborne infectious diseases may reach a major turning point—from a framework dependent on behavioral restrictions and requests to wear protective devices to 1 grounded in voluntary control of individual exposure.

## A Structural Oversight: Why Has an Apparently Obvious Option Never Been Evaluated?

The filtration performance of HEPA filters, the prevention of ambient-air ingress through positive pressure, and the dose-based understanding of infection described here are not new principles; each is grounded in existing research and standards [11,20,22].

No controlled trial has measured the effect of population-level PAPR use on infection rates, and prospective studies evaluating real-world effectiveness, adherence, and adverse events are scarce [12]. World Health Organization (WHO) preparedness and resilience for emerging threats (PRET) module 1 provides an integrated planning framework for respiratory pathogen pandemics [26]. However, positive-pressure exposure control for the general public is not explicitly identified as an independent candidate intervention. The absence of an institutional conceptual category may help explain why the option has not been evaluated despite the existence of its underlying principles and technology.

PAPRs have primarily been studied and used as respiratory protection for health care workers exposed to highly infectious diseases. The systematic review by Licina et al [12] found that existing PAPR research mainly addressed the protection of health care workers from highly infectious viral diseases and identified the higher cost of PAPRs relative to N95 respirators as an implementation disadvantage. A narrative review by the same research group showed that PAPRs have been considered protective equipment for health care workers performing high-risk procedures—particularly aerosol-generating procedures (AGPs)—on patients with suspected or confirmed COVID-19 pandemic [27]. Elkington et al [28] further reported that existing certified PAPRs typically cost £600 to £1100 (GBP £1=US $1.35, as of August 14, 2026) and explicitly identified this high price as one of the main reasons why PAPRs were not widely adopted worldwide during the COVID-19 pandemic, even among health care workers.

Thus, PAPRs were developed within the conceptual category of high-performance, expensive, specialized protective equipment for health care workers facing a high risk of infection. Face masks, by contrast, were treated as an adjunctive behavioral intervention [29]. The idea of redesigning and evaluating PAPRs—which were not widely adopted even among health care workers—as a population-level infection-control measure for routine use by the general public therefore appears to have remained effectively outside conventional research and institutional assumptions. The concept of a full-fledged engineering exposure-control device worn routinely by the general public fit none of the conventional categories and occupied an academic and institutional no-man’s-land. Without a category, a concept cannot even be placed on the agenda for evaluation.

The cost of this structural omission became real during the COVID-19 pandemic. When an unknown pathogen emerged, rapidly deployable options were effectively limited to behavioral interventions plus vaccination, or lockdown. A third option—one with the potential to reduce inhalation exposure by orders of magnitude regardless of pathogen type—existed in principle but was absent in practice. Closing this gap is precisely what should be done now to prepare for the next pandemic.

PFE should therefore be examined by combining medical and engineering assessments within a public health framework. Engineering assessment should use an APF of 1000 as a high-performance benchmark and establish aerosol-protection performance, maintenance of positive pressure, removal of exhaled carbon dioxide, stability of device performance, and applicable conditions of use in ordinary daily life. Medical assessment should determine how an engineering-measured reduction in exposure translates into inhaled infectious dose and individual-level prevention of infection, while also evaluating the safety of prolonged use. Public health assessment should determine how individual-level preventive effectiveness, use rates, and fit or use status translate into population-level infection-control effectiveness and identify the protection levels and use rates necessary and sufficient to achieve infection-control objectives. Feasibility of implementation, social acceptability, cost, and equity should also be evaluated comprehensively. If networking through PWS-NET or a similar system is used, the effect of the PAPR alone should be distinguished from the additional effect of networking, and the effects on privacy and citizens’ rights should be treated as independent evaluation outcomes.

To translate the rapid deployment feasibility of PAPRs into an actual pandemic response, beginning development only after a crisis occurs would be insufficient. During nonemergency periods, performance requirements, safety, sustained use, and conditions of use for public-oriented models should be evaluated, while the number of units required, manufacturing capacity, standardization of components and consumables, and systems for procurement, stockpiling, and distribution should be considered in advance. Current PAPRs are bulky and may appear excessive in daily life; nevertheless, models certified with an APF of 1000 can provide high aerosol-protection performance under specified conditions of fit and operation. A realistic approach is to prepare practicable models first and then progressively reduce weight and noise and ensure adequate airflow during physical activity on the basis of evaluation and operational experience.

## Conclusions

Another respiratory infectious disease pandemic is likely to occur in the future. The question is whether we will search for new countermeasures only after the next crisis begins or evaluate concrete, testable candidate interventions in advance during the current nonemergency period.

Exposure control using positive-pressure PAPRs for the general public directly reduces aerosol inhalation exposure in the individual breathing zone—the process that precedes airborne infection—regardless of pathogen type. Its principles and technology are already established, and existing engineering and theoretical research has brought it to a stage at which it can be considered a candidate intervention with the potential for manufacture and implementation. Nevertheless, it has not been positioned as a subject of formal public health evaluation as a population-level infection-control measure.

This viewpoint does not advocate the immediate introduction of PFE or a universal mandate for its use. Existing engineering and theoretical evidence does not establish the effectiveness of PFE; rather, it justifies beginning its formal evaluation. Although the roles of engineering, medicine, and public health overlap in practice, engineering assessment should verify aerosol-protection performance, maintenance of positive pressure, removal of exhaled carbon dioxide, and stability of device performance in ordinary daily life conditions. Medical assessment must determine how engineering-measured reductions in exposure translate into individual-level prevention of infection and safety during prolonged use. Public health assessment must examine how individual-level preventive effectiveness, population use rates, and continuous and proper use at the necessary times and locations translate into population-level infection-control effectiveness. Feasibility of implementation, social acceptability, cost, and equity must also be evaluated comprehensively.

An APF of 1000 provides a benchmark for evaluating high individual protection, but the performance required of PAPRs for the general public need not uniformly be an APF of 1000. The protection level that is necessary and sufficient to achieve individual- and population-level infection-control objectives should be determined through public health evaluation incorporating medical and engineering evidence. Networking through PWS-NET or similar systems should also be distinguished from evaluation of the PAPR alone; its additional infection-control effect and its implications for privacy and citizens’ rights must be evaluated independently.

To advance this evaluation institutionally, we propose that WHO and national pandemic preparedness plans explicitly position positive-pressure exposure control for the general public as a “candidate intervention for which evidence of effectiveness needs to be accumulated.” Field studies should begin during nonemergency periods to evaluate engineering performance, individual-level preventive effectiveness, population-level infection-control effectiveness, and implementation conditions in stages, while examining the feasibility of manufacturing, procurement, stockpiling, and distribution. Positive-pressure exposure control for the general public should be added to the scope of formal public health evaluation before the next pandemic arrives.

## Abbreviations

AGP: aerosol-generating procedure
APF: assigned protection factor
GLO: Guide to the Expression of Legitimacy of Output
HEPA: high-efficiency particulate air
MPPS: most penetrating particle size
PAPR: powered air-purifying respirator
PFE: Powered air-purifying respirator for Everyone
PRET: preparedness and resilience for emerging threats
PWM: pulse-width modulation
PWS-NET: Powered air-purifying respirator Wear-Rate Management System Network
SAT: saved allowable time
SWPF: simulated workplace protection factor
VRAIO: Verifiable Record of AI Output
WHO: World Health Organization
WPF: workplace protection factor
